# A Semi-Automatic Method to Create an Affordable Three-Dimensional Printed Splint Using Open-Source and Free Software

**DOI:** 10.7759/cureus.13934

**Published:** 2021-03-16

**Authors:** Zhujiang Wang, Adam Dubrowski

**Affiliations:** 1 Faculty of Health Sciences, Ontario Tech University, Oshawa, CAN

**Keywords:** 3d printed splints, 3d printed casts

## Abstract

Three-dimensional (3D) printed splints are becoming more feasible in recent years, showing promising lightweight, waterproof, and hygienic designs. A typical procedure to create 3D printed splints is obtaining the geometry of a body segment using a 3D scanner, creating a 3D printable splint model based on the geometry of the body segment, and 3D printing the splint. As technologies of 3D scanning and 3D printing become mature gradually, the main challenge to fabricate 3D printed splint is to create 3D printable splint models. To solve this challenge, researchers have proposed various methods to design 3D splint models. However, most methods require extensive 3D modeling skills that medical professionals are lacking. In this work, a semi-automatic method is proposed to create a 3D printable model. Given the geometry of a body segment obtained through a 3D scanner, the method includes three key steps: (1) create a draft splint lattice structure, (2) optimize the splint structure, and (3) create a 3D printable model based on the optimized structure. All the software adopted for this method is free and readily available, and thus, there is no additional cost to convert from a scanned geometry of a body segment to a 3D printable splint model. Because the majority of the work is done automatically, with initial training, a medical professional should be able to create a 3D printable model using the proposed method, given the geometry of a body segment. The proposed method is demonstrated by creating a 3D printed wrist splint and the demo is uploaded into GitHub, a popular open-source platform.

## Introduction

Fabric, plaster, wire, elastomers, and low-temperature thermoplastics are the main materials for creating splints [1]. The traditional fabrication process of custom-made splints has remained nearly untouched since the beginning of its use at the end of the 18th century [2]. This process is skill-dependent, and the splints themselves pose numerous problems concerning patient compliance and plaster cracks [3]. To overcome these problems, researchers and engineers have explored additive manufacturing (AM) to create personalized splints. Although fabricating three-dimensional (3D) printed splints are also skill-dependent, a well systematic design process can ensure 3D printed splints conform to the geometry of the human body segment and thus offer comfort to patients. Three-dimensional printed splints using lattice-structures are very popular since they are lightweight, waterproof, hygienic (does not cause a bad odor), and have better air circulation compared with traditional plaster splints. More importantly, few cases are reporting that patients are allergic to common 3D printing materials, such as polylactide (PLA) and acrylonitrile butadiene styrene (ABS).

For example, the Cortex cast was one of the first splints manufactured using a 3D printing technique [4]. Cortex uses a lattice design to stabilize bone fractures and provide immobilization with enhanced air circulation. Recently, this technique is becoming very popular. Palousek et al. explored a general methodology to create a low-cost 3D printed splint for a wrist orthosis [5]. In this method, a 3D splint model is developed based on a 3D scanned geometry of a wrist and is printed using fused deposition modeling technology. This is one of the few 3D printed splints that does not use the lattice style design. Following a similar method, ActivArmor utilizes AM technology to create personalized splints to ensure full immobilization, which is one of the commercial 3D printed products available in the United States [6]. In the work by Cook et al. and Mavroidis et al., 3D printed splints were successfully created for foot orthoses [7,8]. However, these studies did not discuss the detailed process of creating 3D printable models based on 3D scanned geometry.

The arm splint developed by Blaya et al. is one of the few works that has described the process to design a 3D printable structure in detail [2]. The manufacturing process requires in-depth knowledge of the 3D modeling software. Li and Tanaka proposed a semi-automatic design system to create a 3D printable model using Rhinoceros 3D Version 5.0 (Robert McNeel & Associates, Seattle, WA) with a visual programming tool - Grasshopper 3D (Robert McNeel & Associates); however, the software is not free and requires additional costs to create a splint model [9]. All of these factors can be perceived as barriers to the implementation of AM splints into the health care practice.

Today, as many commercial 3D scanners can be used to obtain the geometry of a body segment, the challenge to obtain the 3D geometry of a body segment is mitigated. As the 3D printing technologies become mature, the main challenge to fabricate 3D printing splints is creating 3D printable models of splints. In this work, we propose a semi-automatic method that can create 3D printable splint models based on 3D scanned geometry of human body segment in STL format. The innovations of our work are (1) the process to create 3D printable models is semi-automatic and requires only minimum knowledge in 3D modeling, which is expected to be mastered by frontline doctors or nurses through a short period of training (less than four hours) or even self-directed learning according to the demo submitted in GitHub (available at https://github.com/researchShare/3D-printed-splints-demo-tutorial) and (2) the software used in this work are free and thus the proposed method does not require additional costs.

## Technical report

**Figure 1:**
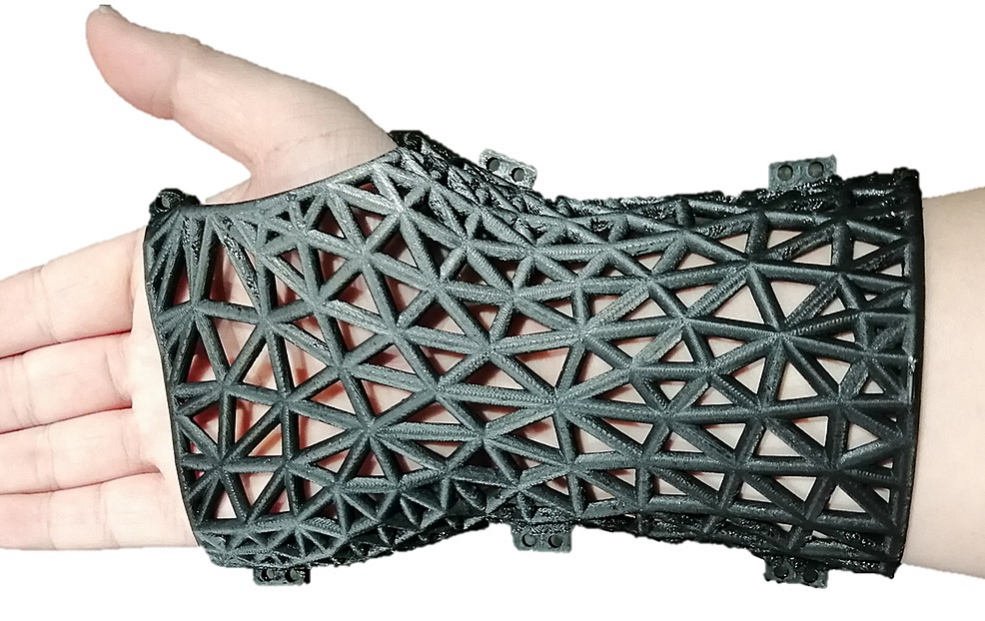
The 3D printed splint perfectly covers the wrist. The radius of the bars composed of the triangular lattice structure is 2 millimeters (mm).

The proposed method to create 3D printable splint models is demonstrated by fabricating a 3D printed wrist splint shown in Figure 1 below.

**Figure 2:**
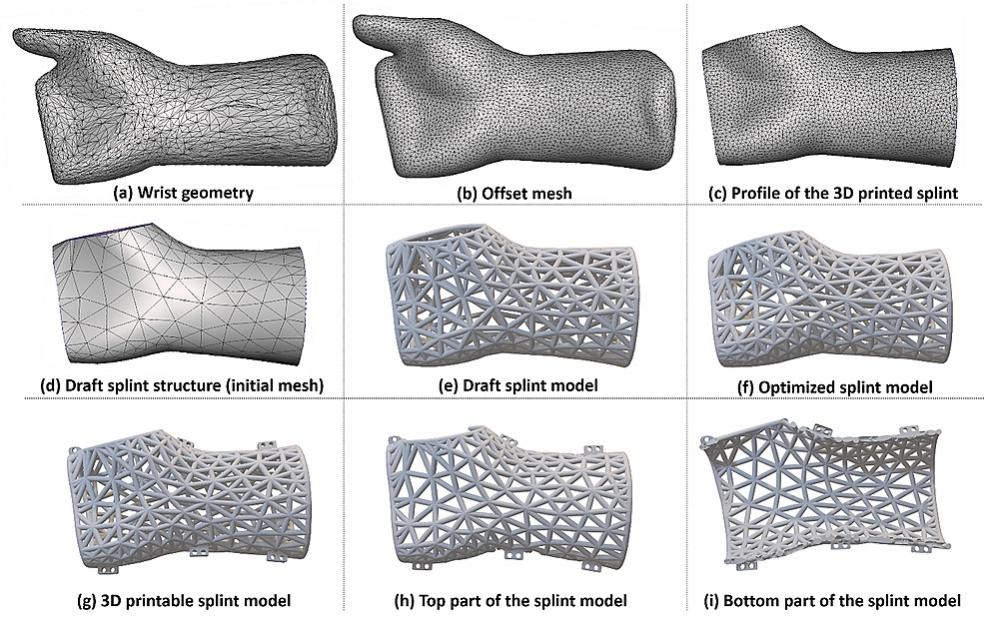
The detailed procedure to create 3D printable splint models based on the geometry of a wrist. (a) The geometry of the wrist is obtained using Artec 3D Spider scanner and Artec Studio 14 Professional software. (b) The mesh represents the offset surface of the wrist geometry with a distance of 2 mm. (c) The profile of the wrist splint is obtained by removing the unnecessary parts of the offset surface. (d) The regenerate mesh (initial mesh) represents the draft splint lattice structure. (e) The draft splint model is created according to the regenerated mesh, and the edges of the regenerated mesh are the centerlines of the bars of the draft splint model. (f) An optimization algorithm is developed to obtain the structure of the optimized 3D splint model. The 3D printable model was created in Blender 2.8 using Python application programming interface (API). (g) Blocks with holes are attached to the 3D splint model, which is separated into two pieces. (h) The top part of the 3D splint model. (i) The bottom part of the 3D splint model. The two pieces splint models can be fixed together around the patients’ wrist through the holes on the blocks. The demo of creating the 3D printable splint is uploaded to GitHub (available at https://github.com/researchShare/3D-printed-splints-demo-tutorial).

In the demo, firstly, we obtained the geometry of the wrist (Figure 2a) using an Artec 3D Spider scanner. Following the detailed procedures shown in Figure 2, a 3D printable splint model was created. Lastly, the wrist splint was printed using Ultimaker S5 3D printers. In the following part, we will discuss the details to fabricate the 3D printed splint. 

Obtaining wrist geometry

The Artec 3D Spider scanner and Artec Studio 14 Professional software were used to obtain the geometry of the wrist, which is represented as the mesh (in STL file format) shown in Figure 2a. The geometry of the wrist can also be obtained through other 3D scanners. As this work focuses on filling the gap between 3D scanned geometry of human body segments and 3D printable splint models, the details of obtaining the wrist geometry using Artec 3D Spider scanner are not going to be discussed here. 

Creating the 3D printable splint model

The method to create a 3D printable wrist model based on the 3D geometry of the wrist is the innovation of this work and includes three major steps: (1) drafting the splint structure, (2) optimizing the splint structure, and (3) designing the 3D printable splint model.

Drafting the Splint Structure

Because the 3D printed splint perfectly covers the wrist (Figure 1) and the radius of the bars composing the wrist splint is set as 2 mm, the centerline of the bars of the wrist splint can form an offset mesh that is 2 mm over the wrist geometry mesh (Figure 2a). In our proposed method, we first obtain the offset mesh (Figure 2b) by creating an offset surface of the wrist geometry mesh (Figure 2a) with a distance of 2 mm (equal to the radius of the bars of the 3D printed splint) through the surface offset function of the free software Meshmixer 3.5 (https://www.meshmixer.com/download.html). Next, the unnecessary parts of the offset mesh are removed using the Meshmixer 3.5 plane cut function, and this results in the profile of the 3D printed splint (Figure 2c). Finally, we use the remesh function of the Meshmixer 3.5 to obtain a new mesh (Figure 2d). The details of creating the draft splint structure using Meshmixer 3.5 are shown in Video 1. Note that the edges of the new mesh are the centerlines of the draft splint model shown in Figure 2e. Therefore, the new mesh in Figure 2d is considered as the draft splint structure. Note that in this work, the new mesh is required to be in OBJ format (a geometry definition file format first developed by Wavefront Technologies) created by Meshmixier 3.5.

**Video 1:** Create the draft splint structure. The video includes the details to convert the wrist geometry (Figure 2a) to the draft splint structure (Figure 2d).

**Figure 3:**
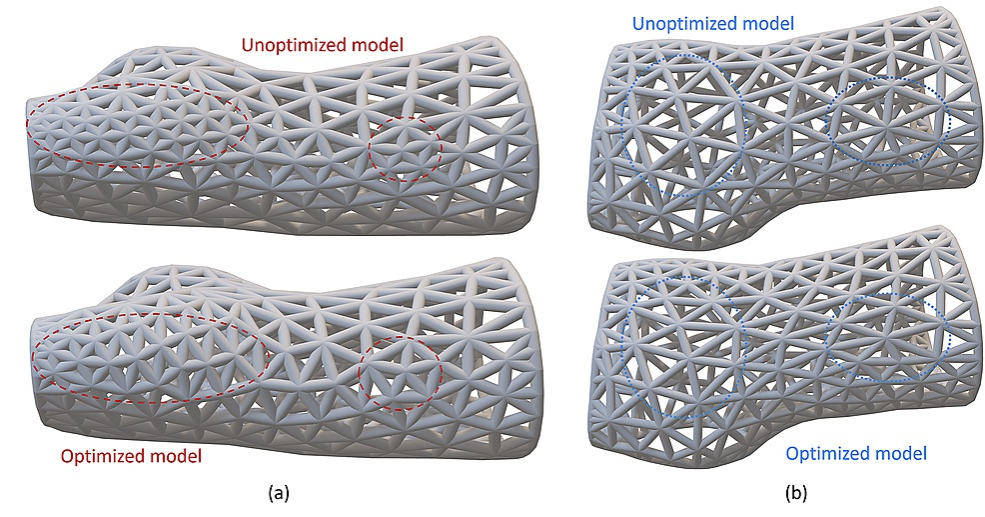
After optimization, (a) the closed triangles highlighted by the dashed circle have become open, and therefore the air circulation around that area is improved. (b) The larger triangles highlighted by the dotted circle become smaller, thus strength is improved around this area.

However, in many cases, the air circulation of a lattice structure directly created through Meshmixer 3.5 cannot be guaranteed. For example, the unoptimized splint model of Figure 3a shows different views of the 3D splint model shown in Figure 2e. The parts highlighted in dashed circles of the unoptimized splint model are too dense, and thus the air circulation may be compromised. Therefore, the structure needs to be optimized to ensure good air circulation.

Optimizing the Splint Structure

To optimize the splint lattice structure represented by the initial mesh in Figure 2d, we develop an optimization algorithm based on the mass-spring system employed in DistMesh [10]. Since a pure mass-spring system can cause continuous vibrations, momentum-based viscous forces are introduced to stabilize the optimization process (see the details of the mass-spring system-based optimization algorithm in the Appendices) [11]. Such a mass-spring system-based algorithm can reduce the size of large triangles and increase the size of small triangles of a mesh. As shown in Figures 2f, 3, it is obvious that the small holes have become bigger, and therefore the air circulation around that area is improved. Additionally, the large triangular holes become smaller, and thus the strength of the splint is improved around this area. The optimization algorithm has been packed into software for Windows 10 system and uploaded into GitHub. Healthcare professionals can use it without requiring any knowledge of programming (Video 2). Note that the optimization algorithm requires initial mesh in the OBJ format exported from Meshmixer 3.5 at present.

**Video 2:** Optimize the draft splint structure. The optimization algorithm has been packed into software uploaded in GitHub.

Designing the 3D Printable Splint Model

The goal of this work is to remove the complex, tedious, and time-consuming 3D-modeling process for designing 3D printable splint models. In this step, we develop an automatic 3D modeling method based on the open-source 3D modeling software Blender 2.8 (https://www.blender.org/download/releases/2-80/). To achieve this goal, a Python script is created to read the structure information of the optimized splint lattice structure obtained in the previous section and draw spheres and cylinders using Python API provided by Blender 2.8 (Video 3). The positions of the spheres are the same as the positions of vertices and the diameter is set to 4 mm. The centerlines of the cylinders are the same as the edges and the diameter of each cylinder is set to 4 mm. The resulting 3D model is shown in Figure 2f.

**Video 3:** Create 3D splint model. The video includes the details to create the optimized splint model (shown in Figure 2f) using Blender 2.8 and Python Code. Note that users do not need to have any knowledge of Python code.

Once the optimized splint model (Figure 2f) was constructed, six predefined blocks were manually attached to the optimized splint model (Video 4). These blocks include small holes to help secure the splint to the wrist. The resulting 3D printable splint model is shown in Figure 2g. In this step, a user needs to learn basic operations about moving objects to attach the blocks to the splint model in Blender 2.8, and these operations are the only modeling techniques that a user should master. As such modeling techniques are very simple, a healthcare professional can design 3D printable splint models using the proposed method in this work after a short period of training. 

**Video 4:** Attach holders. The video includes the details to attach holders manually to the optimized splint model (Figure 2f) to obtain the 3D printable splint model (Figure 2g). A user only needs to learn basic operations (moving objects) in Blender 2.8.

To enable patients with ease, the 3D model is manually separated into two pieces (Figures 2h, 2i) using the plane cut function in Meshmixer 3.5 (Video 5). The two pieces can then be fixed together through the holes on the six blocks. 

**Video 5:** Separate the 3D splint model into two pieces. The video includes the details to separate the 3D splint model into two pieces using Meshmixer 3.5 to obtain the top and bottom part of the 3D printable models in Figures 2h, 2i.

Printing the splint

**Figure 4:**
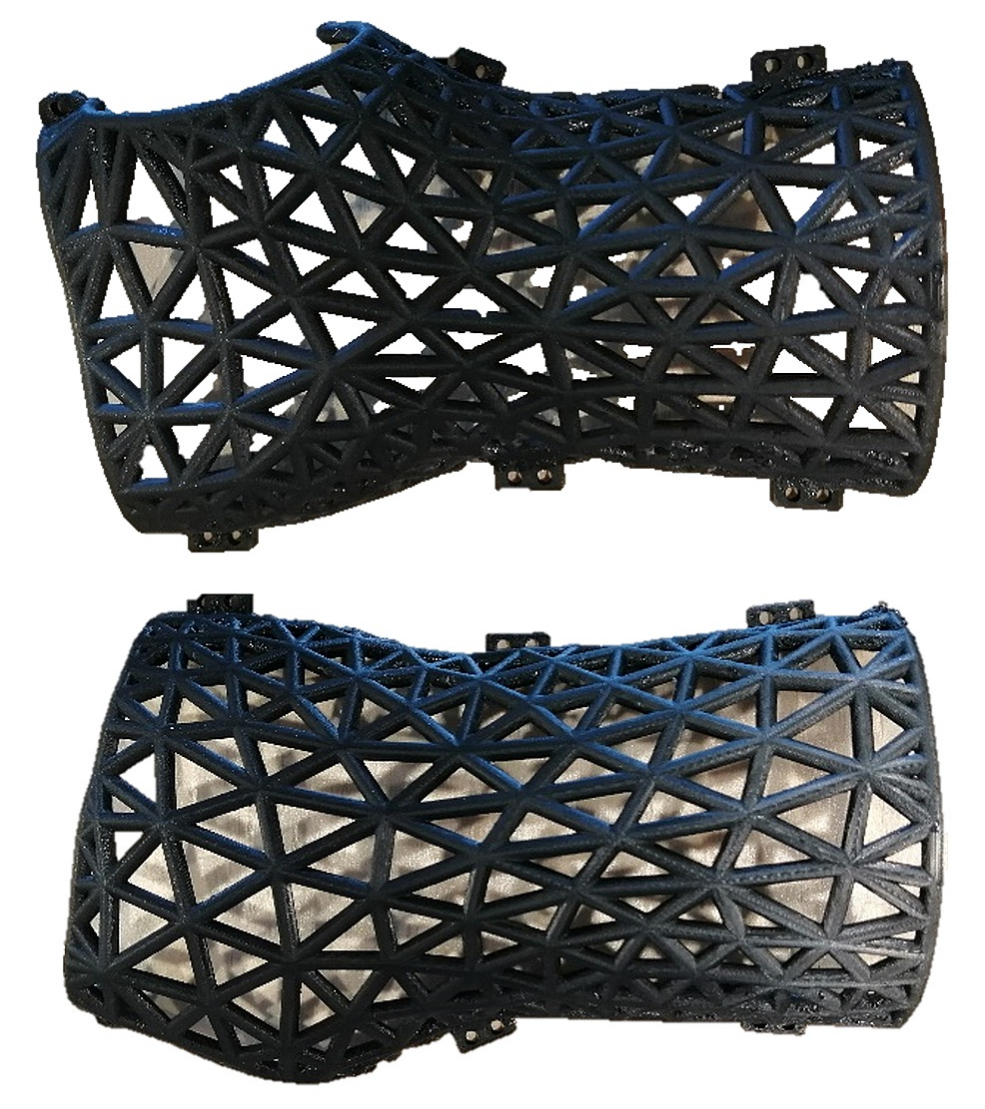
The splint was printed by an Ultimaker S5 using black PLA filaments. The total weight of the two pieces is 126 grams. The material cost of the splint is about $8.00.

The splint was 3D printed on two Ultimaker S5 3D printers (each piece per printer) using black PLA filaments. The material cost was about $8.00 and the printing time was approximate 12 hours (0.2 mm/layer, 100% infills). The 3D printed splint is shown in Figure 4. Such a splint can be printed using other materials and 3D printers. Currently, 12 hours of printing time on an Ultimaker S5 3D printer may limit the applications of the 3D printed splints. However, with the development of 3D printing technologies, the printing speed can be reduced, and the printing time will be no longer a limitation. As the focus of this work is on create 3D printable splint models, the details about the printing tasks are not discussed here.

## Discussion

A systematic method is proposed to create a 3D printable splint model based on truss structures. To validate the method, we fabricate the 3D printed splint for a wrist (Figure 1), and the detailed procedure is uploaded in GitHub as a demo/tutorial. The majority of the modeling tasks to create the 3D printable splint model are done automatically. The major manual tasks include creating the draft splint structure in Meshmixer 3.5, attaching blocks to the 3D splint model in Blender 2.8, and separating the model with blocks into two pieces in Meshmixer 3.5. Among these tasks, attaching blocks manually to 3D splint models in Blender 2.8 is the most complicated one, which requires very basic knowledge of 3D modeling in Blender 2.8, such as moving objects. Therefore the entire procedure can be master by a user (without knowledge of 3D modeling) after a short period of training (less than four hours) or even through self-directed learning based on the demo uploaded in GitHub. The 3D printed splint is waterproof, low-weight (compared to traditional plaster splints/casts), hollow, and strong, and proper 3D printing materials can also avoid allergy. As the air circulation of some draft splint structures created by Meshmixer 3.5 is not always good (Figure 3), an optimization algorithm, which has been packed into software for Windows 10 system and uploaded into GitHub, is created to mitigate the issue. The optimization algorithm can open closed holes, and therefore improve the air permeability. Although the proposed method uses Python 3.7 to draw 3D models in Blender 2.8 and optimize the draft splint structure, users do not need to know the programming language, because the optimization algorithm is packed into software and the Python code for drawing 3D models automatically in Blender 2.8 is uploaded in GitHub. Since all the software utilized was either open-source and free, there is no additional cost to creating the proposed 3D splint model, given a scanned geometry of a body segment. It is worth noting that the same process can be used to create personalized 3D printed splints for any part of a body.

## Conclusions

The recent advances in 3D printing technologies are revolutionizing the fabrication of 3D printed splints. As the technologies of 3D scanners are becoming mature, scanning a human body segment’s geometry is no longer a big challenge. However, frontline doctors and nurses are struggling with converting 3D models to 3D printable models. In this work, a systematic, semi-automatic method is proposed to create a 3D printable splint model using open-source and free software. This method only requires a minimum of 3D modeling knowledge which can be mastered after a short tutorial. Also, the method requires no additional software cost to convert a 3D geometry of the human body segment to a 3D printable splint model. The fabrication of the wrist splint shown here was approximate $8.00 in materials and 15 minutes of designer time. In summary, our work suggests that by using commercially available surface scanners and open-source and free software, it is possible to build a low-cost, fully customizable splint.

However, the proposed method still has two limitations: the method still requires 3D modeling knowledge, and there is no metric on the air circulation and strength of a splint. In the future, we will test the application of the method in a clinical environment. Furthermore, we will simplify the process to reduce human intervention to create 3D printed splints, so that users do not need to have any knowledge of Blender 2.8. We will also conduct research on the materials used for printing splints and analyze splint structures to ensure air circulation and the strength of a splint. Additionally, the x-ray transparent properties of 3D printed splints will be studied in the future as this will allow patients to be examed without removing splints. 

## Figures and Tables

**Figure 5:**
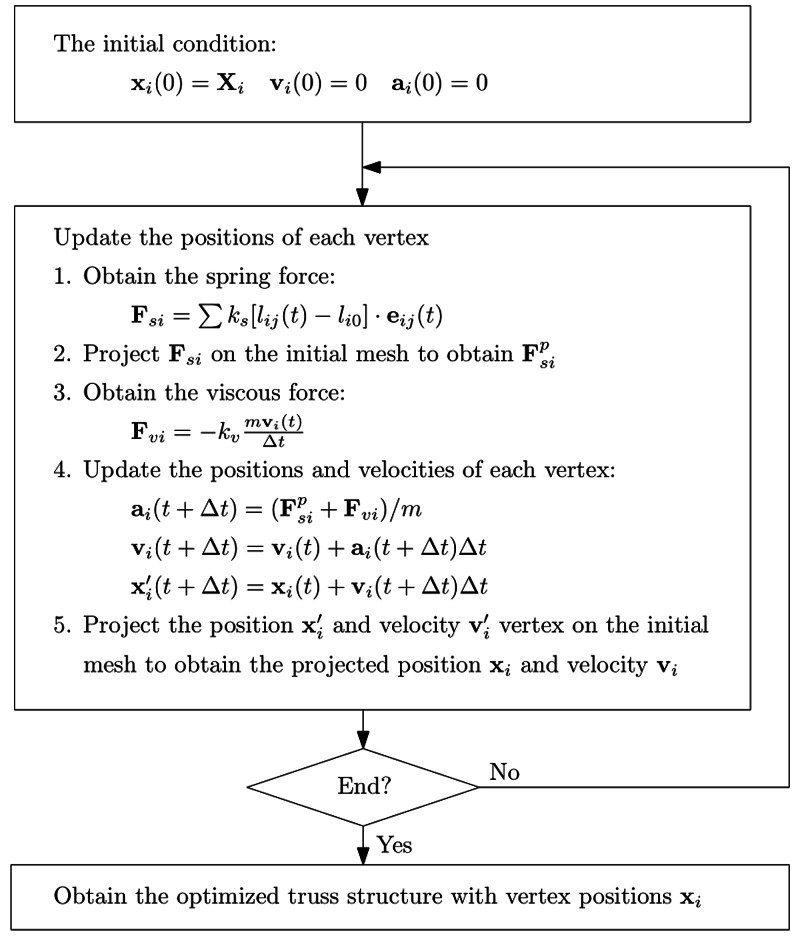
Overview of the optimization algorithm.

**Figure 6:**
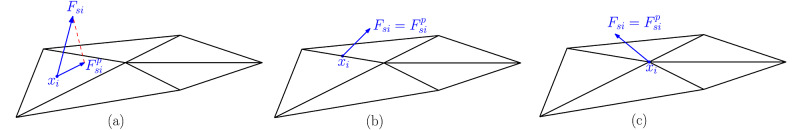
The figure shows the methods to project the spring forces onto the mesh surface. Represented by Figure 2d.

**Figure 7:**
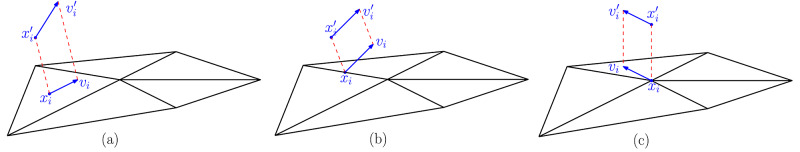
The methods to project the vertices' positions and velocities.

## Appendices

The mass-spring system-based optimization algorithm

The overview of the optimization algorithm is shown in Figure 5. At the initial time step ($t=0$ s), the position $\mathbf{x}_i (0)$ of the $i_{th}$ vertex of the structure is equal to the initial position $\mathbf{X}_i$  ($\mathbf{x}_i (0)=\mathbf{X}_i$), $i=1,2,\cdots, N$, where $N$ is the total number of the vertices of the mesh in Figure 2d. The velocities and accelerations of the vertices are all zero ($\mathbf{v}_i (0)=0$, $\mathbf{a}_i (0)=0$); to better explain the algorithm, the configuration of the regenerated mesh in Figure 2d is named as an initial mesh.

An explicit time method is employed to update the positions of the vertices. $\Delta t$ is the size of each time step. The optimization process includes five steps.  The first step is calculating the spring force $\mathbf{F}_{si}$ applied on the $i_{th}$ vertex

$\mathbf{F}_{si}=\sum_{j=1}^{N_i} k_s [l_{ij} (t)-l_{i0} ]\mathbf{e}_{ij} (t)$

where $\mathbf{e}_{ij} (t)=\frac{\mathbf{x}_i (t)-\mathbf{x}_j (t)}{|\mathbf{x}_i (t)-\mathbf{x}_j (t)|}$; $\mathbf{x}_i (t)$ is the position of the $i_{th}$ vertex; $\mathbf{x}_j (t)$ the position of the $i_{th}$ vertex’s neighbor vertex, which is connecting with the vertex $\mathbf{x}_i$ through an triangle edge; $l_{ij} (t)=|\mathbf{x}_i (t)-\mathbf{x}_j (t)|$ the length of an edge with two end vertices $\mathbf{x}_i$ and $\mathbf{x}_j$; $l_{i0} = \frac{c}{N_i} \sum l_{ij} (0)$ the average length of the edges containing the vertex $\mathbf{x}_i$ at initial time step; $N_i$ the total number of the $i_{th}$ vertex’s neighbor vertices; $c$ a constant (in this work, we set $c=0.5$, and all the springs are assumed in stretching state at the initial time step); $k_s$ the spring constant. 

To keep the profile of the mesh in Figure 2d, the vertices $\mathbf{x}_i (t)$ are only allowed to move on the surface of the initial mesh. The direction of the spring force $\mathbf{F}_{si}$ can be outside of the plane of the triangle where the $i_{th}$ vertex is located, so $\mathbf{F}_{si}^p$ is considered as the projection of the force $\mathbf{F}_{si}$ on the vertex’s current location. As shown in Figure 6a, when the vertex $\mathbf{x}_i$ is inside of a triangle of the initial mesh, $\mathbf{F}_{si}^p$ is the projection of the force $\mathbf{F}_{si}$ on the triangle. When the vertex $\mathbf{x}_i$ is on an edge of the mesh or the position of the vertex $\mathbf{x}_i$ is the same as the position of a vertex of the initial mesh (Figures 6b, 6c), the projected force $\mathbf{F}_{si}^p$ is equal to $\mathbf{F}_{si}$ ( $\mathbf{F}_{si}^p=\mathbf{F}_{si}$). 

Since a pure mass-spring system can cause continuous vibrations, viscous forces $\mathbf{F}_{vi}$ is introduced as $\mathbf{F}_{vi} = -k_v\frac{m \mathbf{v}_{i}(t)}{\Delta t}$, where $k_v$ is a viscous constant. The positions of the vertices are updated based on the Euler method through the following governing equation

$m \mathbf{a}_i (t + \Delta t) =\mathbf{F}_{si}^p+\mathbf{F}_{vi} \\ \mathbf{v}_i (t + \Delta t) =\mathbf{v}_i (t)+\mathbf{a}_i (t + \Delta t)\Delta t \\ \mathbf{x}_i'(t + \Delta t) =\mathbf{x}(t)+\mathbf{v}_i (t + \Delta t)\Delta t$

where $m$ is the mass of each vertex.

As shown in Figure 7, if a vertex's position $\mathbf{x}_i '(t + \Delta t)$  is off the initial mesh surface, we project the vertex's position  $\mathbf{x}_i '(t + \Delta t)$  and velocity  $\mathbf{v}_i '(t + \Delta t)$ onto the initial mesh surface and obtain the updated vertices' positions  $\mathbf{x}_i(t + \Delta t)$ and velocities $\mathbf{v}_i '(t + \Delta t)$ at the next time step. The projected point $\mathbf{x}_i (t + \Delta t)$ is such a point on the initial mesh that $d= |\mathbf{x}_i '-\mathbf{x}_i |$ is the shortest distance between the $\mathbf{x}_i'(t + \Delta t)$ and the initial mesh. If the point $\mathbf{x}_i (t + \Delta t)$ is inside a triangle of the initial mesh (Figure 7a), the velocity is also projected onto the plane of the triangle ($\mathbf{v}_i$ is the projected velocity); if the point $\mathbf{x}_i (t + \Delta t)$ is on an edge of the mesh (Figure 7b) or locates at a vertex of the mesh (Figure 7c), the velocity remains unchanged ($\mathbf{v}_i = \mathbf{v}_i'$). The projected positions and velocities are then considered as the new positions and velocities of vertices at the time step $t + \Delta t$.

Finally, a criterion is created to determine whether the optimization process should be terminated. Assume that $D(t)=\sum _{i=1}^{N} |\mathbf{x}_i (t + \Delta t)-\mathbf{x}_i (t)|$ is the total distance that all the vertices have traveled from the time step $t$ to $t + \Delta t$; $D_{All}^{max} = \max   \left\lbrace  D(\Delta t), D(2\Delta t),\cdots D(t),D(t+\Delta t) \right\rbrace$ is the maximum value among the distances in all the previous time steps. If $\frac{D(t) }{D_{All}^{max} }  &lt; 5 \%$, the vertices are assumed to be stable and the optimization process is terminated. 
